# Silver Nanocomposite Biosynthesis: Antibacterial Activity against Multidrug-Resistant Strains of *Pseudomonas aeruginosa* and *Acinetobacter baumannii*

**DOI:** 10.3390/molecules21091255

**Published:** 2016-09-20

**Authors:** Klebson Silva Santos, Andriele Mendonça Barbosa, Luiz Pereira da Costa, Malone Santos Pinheiro, Maria Beatriz Prior Pinto Oliveira, Francine Ferreira Padilha

**Affiliations:** 1 Institute of Technology and Research, Tiradentes University, Av. Murilo Dantas 300, 49032-971 Aracaju, SE, Brazil; klebson-biomedico@hotmail.com (K.S.S.); andrielemendonca@yahoo.com.br (A.M.B.); lupeco7@hotmail.com (L.P.d.C.); malonepinheiro@hotmail.com (M.S.P.); fpadilha@yahoo.com (F.F.P.); 2 REQUIMTE/LAQV, Department of Chemistry Sciences, Faculty of Pharmacy, University of Porto, R. Jorge Viterbo Ferreira 228, 4050-313 Porto, Portugal; 3 CAPES Foundation, Ministry of Education of Brazil, 70040-020 Brasília, DF, Brazil

**Keywords:** silver nanocomposite biosynthesis, antibacterial activity, multidrug-resistance, *Pseudomonas aeruginosa*, *Acinetobacter baumannii*

## Abstract

Bacterial resistance is an emerging public health issue that is disseminated worldwide. Silver nanocomposite can be an alternative strategy to avoid Gram-positive and Gram-negative bacteria growth, including multidrug-resistant strains. In the present study a silver nanocomposite was synthesized, using a new green chemistry process, by the addition of silver nitrate (1.10^−3^ mol·L^−1^) into a fermentative medium of *Xanthomonas* spp. to produce a xanthan gum polymer. Transmission electron microscopy (TEM) was used to evaluate the shape and size of the silver nanoparticles obtained. The silver ions in the nanocomposite were quantified by flame atomic absorption spectrometry (FAAS). The antibacterial activity of the nanomaterial against *Escherichia coli* (ATCC 22652), *Enterococcus faecalis* (ATCC 29282), *Pseudomonas aeruginosa* (ATCC 27853) and *Staphylococcus aureus* (ATCC 25923) was carried out using 500 mg of silver nanocomposite. *Pseudomonas aeruginosa* and *Acinetobacter baumannii* multidrug-resistant strains, isolated from hospitalized patients were also included in the study. The biosynthesized silver nanocomposite showed spherical nanoparticles with sizes smaller than 10 nm; 1 g of nanocomposite contained 49.24 µg of silver. Multidrug-resistant strains of *Pseudomonas aeruginosa* and *Acinetobacter baumannii*, and the other Gram-positive and Gram-negative bacteria tested, were sensitive to the silver nanocomposite (10–12.9 mm of inhibition zone). The biosynthesized silver nanocomposite seems to be a promising antibacterial agent for different applications, namely biomedical devices or topical wound coatings.

## 1. Introduction

Bacterial infections affect millions of people around the world, being an important cause of morbidity and mortality and having a relevant impact on the healthcare economy [1,2]. The treatment of these infections has become a public health concern due to bacterial resistance to antibiotics [3,4]. *P. aeruginosa* and *A. baumannii* are the major nosocomial pathogens, responsible for healthcare-associated infections and hospital outbreaks, with multidrug resistance to antimicrobial agents such as cephalosporins, fluoroquinolones, aminoglycosides, polymyxins and even carbapenems [2]. A possible strategy to control the resistant microorganisms is to improve the biocide substances activity against them [5].

Recent studies have shown that bacterial resistance to antibiotics spreads more quickly than the development of antimicrobial agents for their combat [4]. Thus, the interest in the synthesis of new products with antimicrobial potential is continuously increasing [6]. This is the case of silver, a metal with a described antibacterial activity and significant efficacy against Gram-negative and Gram-positive bacteria, as well as its silver nanoparticles (AgNPs) [7]. This research area has becoming a challenge for nanomedicine and biotechnology [8]. The bactericidal and bacteriostatic activities of AgNPs are due to its ability to lyse the bacterial cell wall, allowing the exit of the cytoplasm content, inhibiting the respiratory chain, and consequently having injurious effects on the DNA [9]. *Escherichia coli*, *Staphylococcus aureus*, *Bacillus subtilis*, *Salmonella typhimurium*, *Pseudomonas aeruginosa* and *Klebsiella pneumoniae* are pathogenic bacteria sensitive to silver and its nanoparticles [10,11].

According to Soni and Prakash, antimicrobial AgNPs can be synthesized by bacteria as a new green chemistry process [12]. Besides that referred above, AgNPs in conjunction with biopolymers can originate nanocomposites. These nanomaterials also have important antibacterial activity for both Gram-positive and Gram-negative bacteria, emerging as promising alternatives for this issue. Cao et al. described an antimicrobial activity with a broad spectrum of action of AgNPs with chitosan and xanthan gum [13]. In this case, the complete inhibition of *Escherichia coli* and *Staphylococcus aureus* growth was described [14].

Xanthan gum is a biodegradable polymer produced by *Xanthomonas* spp. in a fermentation process, with paramount importance for the food and pharmaceutical industries, as a dispersing agent and gelling and emulsion stabilizer [15,16]. This biomaterial is used in biomedical research, in combination with synthetic drugs, to improve their potential in treatment against infections [17,18]. In this way, the development and assessment of silver nanocomposites as antibacterial agents is of the utmost importance for clinical microbiology as previously mentioned. Moreover, it can contribute to the development of new antimicrobial agents, impregnated in xanthan gum, with a promising effect on multidrug-resistant bacteria.

The aim of the present study was to evaluate the antimicrobial effect of a silver nanocomposite biosynthesized by *Xanthomonas* spp. in a one-step synthesis methodology, against Gram-positive and Gram-negative bacteria and multidrug-resistant strains of *P. aeruginosa* and *A. baumannii*. The use of a single step synthesis methodology should be emphasized as the main novelty of the present study, being economical and time-saving for the industry.

## 2. Results and Discussion

### 2.1. Characterization of the Silver Nanocomposite Biosynthesized

**Figure 1:**
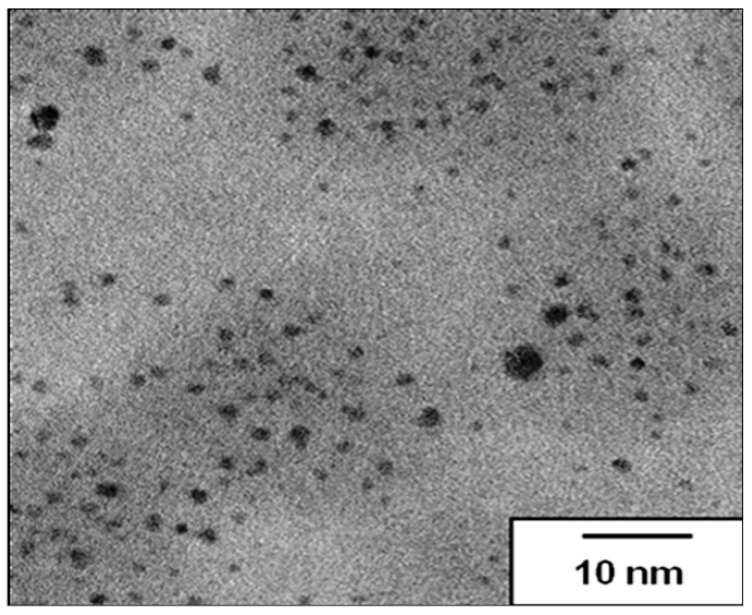
Transmission electron micrograph of silver nanocomposite, stabilized with XG, showing spherical-shape nanoparticles in the polymer.

The biosynthesis by *Xanthomonas* spp. of the silver nanocomposite was obtained by a one-step process, by the addition of silver nitrate into the fermentative medium. As it is possible to observe in Figure 1, the synthesized silver nanoparticles had a size approximately three times smaller than 10 nm and a uniform spherical shape.

Different authors synthesized similar AgNPs by the addition of silver nitrate to xanthan gum, however using a two-step methodology [14,19]. This means that in a first step xanthan gum is obtained, and in a second step the AgNPs are produced. In the present study the AgNPs were obtained in a single step, simultaneously with the xanthan gum. The synthesis was simply performed by the addition of silver nitrate (1.10^−3^ mol·L^−1^) to the fermentative medium where xanthan gum was produced by *Xanthomonas* spp. This innovation can improve the industrial potential of the nanocomposite. According to Palaniraj and Jayaraman, xanthan gum is industrially produced by bacterial fermentation and is used to stabilize suspensions of drugs such as dextromethorphan and thiabendazole [20].

The biosynthesis of AgNPs, with sizes between 5–40 nm and ~9.1 nm, and their stabilization with xanthan gum, had already been reported by Xu et al. and Emam and Zahran, respectively [14,21]. The present results indicate a more efficient biosynthesis of AgNPs due to their sizes, which are smaller than in the literature cited above. Moreover, they are also smaller than the AgNPs synthesized by some fungi, plants or even by chemical approaches [22]. According to Agnihotri et al. AgNPs with a size of 5 nm showed a more powerful antibacterial activity than the ones with 7, 10, 15, 20, 30, 50, 63, 85 and 100 nm average sizes [23]. In fact, the AgNPs with a small size exhibit a higher contact surface area than larger size AgNPs, resulting in a higher antimicrobial activity with a lower concentration of nanosilver.

The quantification of silver ions by flame atomic absorption spectrometry, an accurate and confident method, allowed knowing their levels in the nanocomposite [24]. The silver amount in the nanocomposite is an important issue for future applications of the nanomaterial as an antimicrobial agent. The silver content determined in the present study was 49.24 µg per g of nanocomposite. According to different authors, AgNPs at a concentration lower than 50 µg/mL have a potential inhibition against Gram-positive and Gram-negative strains [25,26].

In fact, the AgNPs’ concentration, shape and size are fundamental to understanding the antimicrobial mechanisms [26]. Palanisamy et al. tested silver nanoparticles (2.5, 5, 10 and 20 µg/mL) against bacterial growth and reported that multidrug-resistant bacteria were more sensitive to AgNPs at 20 µg/mL [27]. Additionally Wilding et al. reported that the oral administration of AgNPs at 1 mg/mL and 10 µL/g of body mass, in repeated doses during 28 days, do not cause a significant effect on the murine gut microbiome [7]. Contrarily, the broad-spectrum antibiotics have more pejorative effects. This information is another advantage of this type of antimicrobial agent, allowing consideration of its incorporation in edible products [28]. Taking into account the characteristics of the nanocomposite biosynthesized, it seems to be a promising antimicrobial agent.

### 2.2. Antibacterial Activity of the Silver Nanocomposite

As previously reported, the antibacterial activity of the silver nanocomposite against Gram-positive and Gram-negative bacteria was evaluated. Table 1 shows the sensitivity of all bacteria tested against the silver nanocomposite.

**Figure 2:**
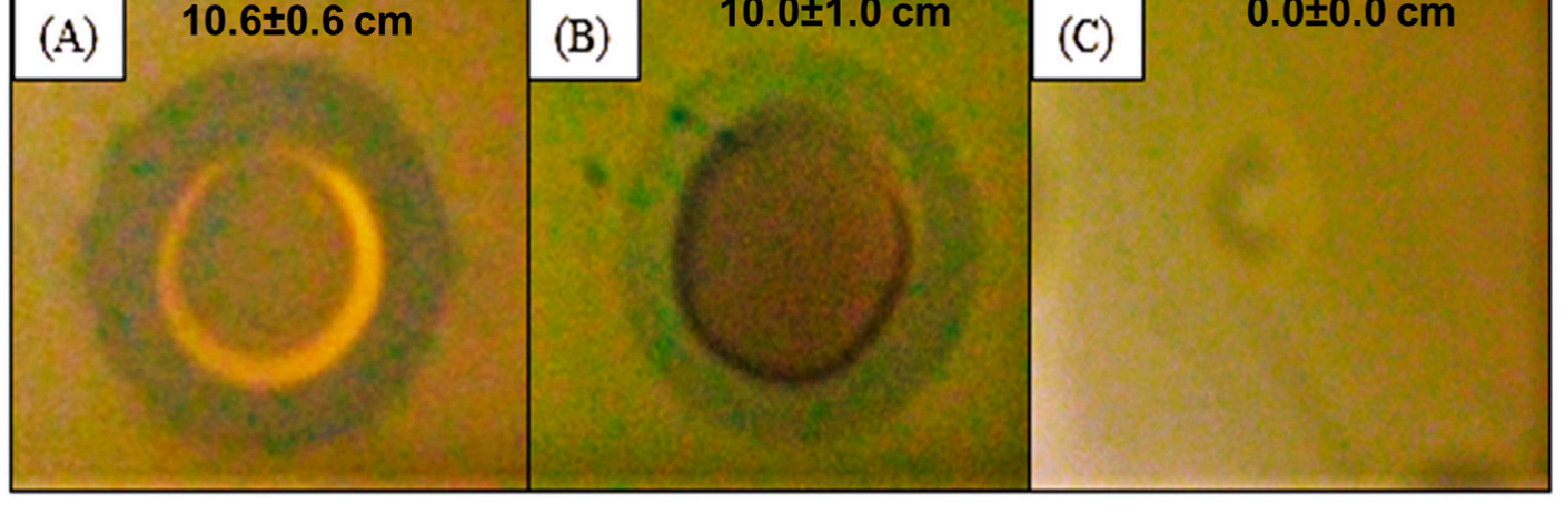
Inhibition zone pictures of silver nanocomposite against *A. baumannii* (**A**) and multi-resistant *P. aeruginosa* (**B**); Xanthan gum without silver nanoparticles was also tested against the cited bacteria (**C**).

The silver nanocomposite (500 mg with 24.62 µg of silver) shows broad-spectrum antibacterial action against *E. coli*, *E. faecalis*, *P. aeruginosa* and *S. aureus.* The effect of AgNPs against Gram-negative and Gram-positive bacteria can be interesting in the treatment, prophylaxis and control of infectious microbial diseases. Moreover, these properties also affect multidrug-resistant microorganisms, according to our results and other published works [23,25,28]. As shown in Figure 2, the silver nanocomposite inhibited the growth of *A. baumannii* and *P. aeruginosa* multidrug-resistant clinical strains.

As shown, the silver nanomaterial synthesized by *Xanthomonas* spp. seems to be effective against several bacteria including multidrug-resistant strains (Table 1). The antibacterial activity can be justified by the small size (less than 10 nm), the spherical shape (Figure 1) and the concentration (24.62 µg Ag/500 mg nanocomposite) of the silver nanoparticles. Several action mechanisms of these nanoparticles, such as modification of the cell wall, increased cytoplasm and membrane permeability, morphological changes, separation of the cytoplasmic membrane from the cell wall, plasmolysis, inhibition of the respiratory activity, and inhibition of bacterial DNA replication or modification of intracellular ATP levels, cause irreversible damage of bacterial cells [29].

The antibacterial effect of the silver nanocomposite can be a promising alternative to avoid the growth of the multidrug-resistant clinical strains, namely *P. aeruginosa* and *A. baumannii*. Similar results were already reported for *P. aeruginosa* [30] and *A. baumannii* [31,32]. Moreover, this nanomaterial showed a similar action on the growth inhibition of multidrug-resistant bacteria presented by AgNPs synthesized by chemical and biological routes [33].

The use of nanotechnology for the production of antibacterial agents aiming to control and treat bacterial infections has already been applied [34,35,36,37], corroborating our results. The silver nanocomposite developed can be an alternative to conventional antibiotics, with special importance in multidrug-resistant bacteria [38,39]. According to several studies, antimicrobial agents with clinical and economic impacts are needed to overcome the global threat of antimicrobial resistance [23,25].

## 3. Materials and Methods

### 3.1. Silver Nanocomposite Biosynthesis

Xanthan gum was obtained by a fermentation process of *Xanthomonas* spp. (cell concentration about 10^11^ CFU·mL^−1^) in 250 mL Erlenmeyer flasks with 86 mL of fermentation medium, according to Rottava et al. [16], and containing (g·L^−1^): MgSO_4_·7H_2_O—0.2; KH_2_PO_4_—5.0; H_3_BO_3_—0.006; (NH_4_)_2_SO_4_—2.0; FeCl_3_—0.0024; CaCl_2_·2H_2_O—0.002; ZnSO_4_—0.002; and sucrose—50.0. In order to obtain the silver nanocomposite, silver nitrate (1.10^−3^ mol·L^−1^) was added to the fermentation medium, in the dark, in an orbital shaker at 200 rpm and 28 °C, during 96 h.

### 3.2. Flame Atomic Absorption Spectrometry (FAAS)

A Perkin Elmer model Analyst 300 flame atomic absorption spectrometer was used to determine the silver ions concentration into the nanocomposite, at 328.1 nm. For the quantitative analysis, standard silver solutions were prepared and a calibration curve plotted among 0–1000 µg/mL. For the determination, 1 g of silver nanocomposite was diluted into 1 mL deionized water.

### 3.3. Transmission Electron Microscopy (TEM)

The morphology and size of silver nanoparticles were determined by Transmission Electron Microscopy (TEM) on a JEOL HTP 2100 instrument, operating at 200 kV. The silver nanocomposite was sonically suspended in deionized water for 1 h and dropped into carbon-coated copper grids [19].

### 3.4. Antimicrobial Activity Assay

*S. aureus* (ATCC 25923), *P. aeruginosa* (ATCC 27853), *E. faecalis* (ATCC 29282) and *E. coli* (ATCC 22652) were used in the study. Isolates of *P. aeruginosa* and *A. baumannii* clinical strains from patients hospitalized in Aracaju, Sergipe, Brazil, were also involved in the study. Strains were identified by Vitek^®^ 2 system (bioMérieux, Marcy-l’Étoile, France). The multidrug resistance to antibiotics, namely ampicillin, cephalexin, cefapirin, cefotaxime, cefoxitin, ceftazidime, cefoxitin, colistin, piperacillin-tazobactam, gentamicin, imipenem and meropenem, was verified.

The antimicrobial activity of the AgNPs was performed on bacteria suspensions (10^8^), spreading with a sterile cotton swab on the Mueller-Hinton agar plate. Then 50 µL of the solution containing the silver nanocomposite (test) and 50 µL of the solution containing xanthan gum without silver (control) were added separately in wells (5 mm) made with sterilized borer. The test solution was prepared with 500 mg of the nanocomposite in 1 mL of distilled water. Finally, plates were incubated at 37 °C for 24 h. After the incubation period, the diameter of the inhibition zone was recorded. All studies were performed in triplicate.

## 4. Conclusions

This study suggests that the silver nanocomposite obtained in a *Xanthomonas* spp. fermentation process, by one-step methodology, is an effective antimicrobial agent against Gram-positive and Gram-negative bacteria, including the multidrug-resistant *P. aeruginosa* and *A. baumannii* isolates from hospitalized patients.

## Figures and Tables

**Table 1:** Diameter of inhibition zone to bacteria tested against silver nanocomposite.

Bacterial Strain	Zone of Inhibition (mm)	
	XG 500 mg/mL	Ag Nanocomposite 500 mg/mL
*Escherichia coli* (ATCC 22652)	0 ± 0	11.6 ± 0.5
*Enterococcus faecalis* (ATCC 29282)	0 ± 0	10.8 ± 0.5
*Pseudomonas aeruginosa* (ATCC 27853)	0 ± 0	12.9 ± 0.8
*Staphylococcus aureus* (ATCC 25923)	0 ± 0	12.2 ± 0.3
*Acinetobacter baumannii* (MDR)	0 ± 0	10.6 ± 0.6
*Pseudomonas aeruginosa* (MDR)	0 ± 0	10.0 ± 1.0
